# Economic evaluation of expanding inguinal hernia repair among adult males in Sierra Leone

**DOI:** 10.1371/journal.pgph.0003861

**Published:** 2024-12-12

**Authors:** Zin Min Thet Lwin, Abdul Rahman Mansaray, Sam Al-Samman, George Keel, Birger C. Forsberg, Jessica H. Beard, Alex J. van Duinen, Håkon Angell Bolkan, Thomas Ashley, Juuli Palmu, Hertta Kalsi, Hannah Ashley, Jenny Löfgren

**Affiliations:** 1 Department of Molecular Medicine and Surgery, Karolinska Institutet, Stockholm, Sweden; 2 Department of Learning, Informatics, Management and Ethics, Karolinska Institutet, Stockholm, Sweden; 3 Department of Global Public Health, Karolinska Institutet, Stockholm, Sweden; 4 Department of Surgery, Lewis Katz School of Medicine at Temple University, Philadelphia, Pennsylvania, United States of America; 5 Department of Surgery, St Olav’s Hospital, Trondheim University Hospital, Trondheim, Norway; 6 Institute of Nursing and Public Health, Norwegian University of Science and Technology, Trondheim, Norway; 7 Department of Surgery, University of Sierra Leone Teaching Hospital, Freetown, Sierra Leone; 8 CapaCare, Sierra Leone; 9 Department of Surgery, Örebro University Hospital, Örebro, Sweden; 10 Department of Surgery, Capio Sankt Görans Hospital, Stockholm, Sweden; 11 The Lakes Medical Practice, Penrith, United Kingdom; Government NSCB Medical College, INDIA

## Abstract

Sierra Leone faces a substantial backlog of patients with inguinal hernia in need of repair due to a shortage of surgical providers. The current mitigation strategy includes task-sharing with associate clinicians and non-specialist medical doctors, and the economic impact of this approach needs assessment for potential scale-up. This study aimed to assess the cost-effectiveness of open mesh repair of inguinal hernias by associate clinicians and non-specialist medical doctors in adult males (>18 years) compared to no treatment, as well as between the two provider types and to estimate the budget impact of clearing the backlog in Sierra Leone. A Markov model was constructed to calculate the cost per disability-adjusted life year (DALY) averted over 10 years for operations by different providers. Subsequently, the costs of reducing the backlog through accelerated repair rates via task-sharing were assessed under two scenarios, with or without a budget limit. Deterministic and probabilistic sensitivity analyses were conducted to evaluate the uncertainty of input values. Associate clinicians and non-specialist medical doctors achieved USD 250 and USD 411 per DALY averted, respectively, which is below the GDP per capita of USD 1,427. Associate clinicians delivered comparable health outcomes at lower costs than non-specialist medical doctors. A budget of USD 108 million was projected to clear the entire backlog over 10 years. Hernia repair by both associate clinicians and non-specialist medical doctors in Sierra Leone is highly cost-effective. Associate clinicians, with quality training and supportive supervision, are more cost-effective than non-specialist medical doctors. Task-sharing, especially with associate clinicians, is promising for optimizing access to surgical services.

## Introduction

Despite significant strides in global health over the past 25 years, the burden of surgical conditions has increased, particularly in low- and middle-income countries (LMICs), where an estimated 94% of the population lacks access to safe, timely, and affordable surgical care [1]. Inguinal hernia repair, an essential surgical procedure, is in high demand [1, 2], especially in Sub-Saharan Africa (SSA), where a large proportion of patients remains untreated [2]. One of the main reasons is a shortage of surgical professionals [3].

Sierra Leone, a low-income country in SSA, has a high incidence and prevalence of inguinal hernia among men. An estimated 8.6% of men have inguinal hernia, and about 1,250 new patients per 100,000 population occur annually [4]. The country also has a significant shortage of surgical providers, with only 0.0625 specialist surgeons per 100,000 population in 2024 [5], falling short of the recommended threshold of 20 per 100,000 population [6]. Consequently, the unmet surgical need was estimated to be approximately 92.7% [7], based on the projected annual surgical need in the country (excluding complications related to the procedures). This shortage has led to a substantial backlog of untreated hernia patients [4].

Task-sharing, recommended by the World Health Organization, offers a cost-effective solution to human resource constraints [1, 8, 9], potentially reducing costs and training time required for scaling up the surgical workforce to meet the recommended threshold by up to 40% nationally [1]. Despite ongoing debates within surgical and anaesthetic communities regarding the safety and efficacy of task-sharing [10, 11], promising outcomes have been observed, especially for inguinal hernia repair [8, 12, 13].

Sierra Leone exemplifies the successful implementation of task-sharing, initiated by CapaCare [12]. In partnership with the Ministry of Health, CapaCare’s training program equips non-specialist medical doctors (MDs) and associate clinicians (ACs) to manage surgical and obstetrical emergencies, increasing the number of skilled staff at district hospitals. A previous nationwide study showed that non-specialists performed 52.8% of all surgeries and 67% of hernia repairs in Sierra Leone [14]. Post-operative mortality rates for non-specialist surgeries ranged from 0.4% to 8.0%, comparable to previous rates in the country and SSA [8]. This success underscores the effectiveness of task-sharing in improving surgical volumes and outcomes.

While task-sharing has demonstrated success in certain contexts, an evidence-based quantification of the cost-effectiveness of task sharing for inguinal hernia repair as compared to standard care remains to be established, particularly when involving ACs and MDs. An economic evaluation is required for evidenced-based health policy and decision-making around effective resource allocation in surgical care. This study aims to determine the cost-effectiveness and budget impact of expanding elective inguinal hernia repair through task-sharing between ACs and MDs in Sierra Leone.

## Materials and methods

### Population and interventions

The study focused on adult males (aged ≥18 years) with symptomatic primary inguinal hernia in Sierra Leone. Both symptomatic and asymptomatic patients were included in the budget impact analysis, assuming that asymptomatic patients would transition to symptomatic status in subsequent years. The investigation assessed the long-term cost and health impact of elective open mesh repair of primary inguinal hernia, performed by MDs and ACs. Children, adult females, and patients requiring emergency surgery were excluded, as these cases are more complex and require closer supervision when performed by non-specialists in a task-sharing context.

MDs were non-specialist medical doctors who completed a six-year medical school and a two-year internship. ACs were mid-level healthcare providers (non-doctors) who completed a 3-year basic medical diploma training. Both MDs and ACs, with ACs requiring at least two years of postgraduate clinical practice, are eligible for the three-year CapaCare training program, which includes surgical training [12].

### Data sources

Data for the study were gathered from prior research in Sierra Leone. Clinical data, including patients’ reported pain levels (before and one year after surgery) and the risk of recurrent hernia one year post-surgery, were obtained from a randomized clinical trial (2017–2019) comparing inguinal hernia repairs performed by ACs and MDs [12]. This was the only study available that assessed the outcomes of hernia repair performed by non-specialists in Sierra Leone. Epidemiological data, such as the prevalence, annual incidence, and repair rates of inguinal hernia, were sourced from the most recent prevalence study conducted in the country in 2020 [4].

### Model structure

**Fig 1:**
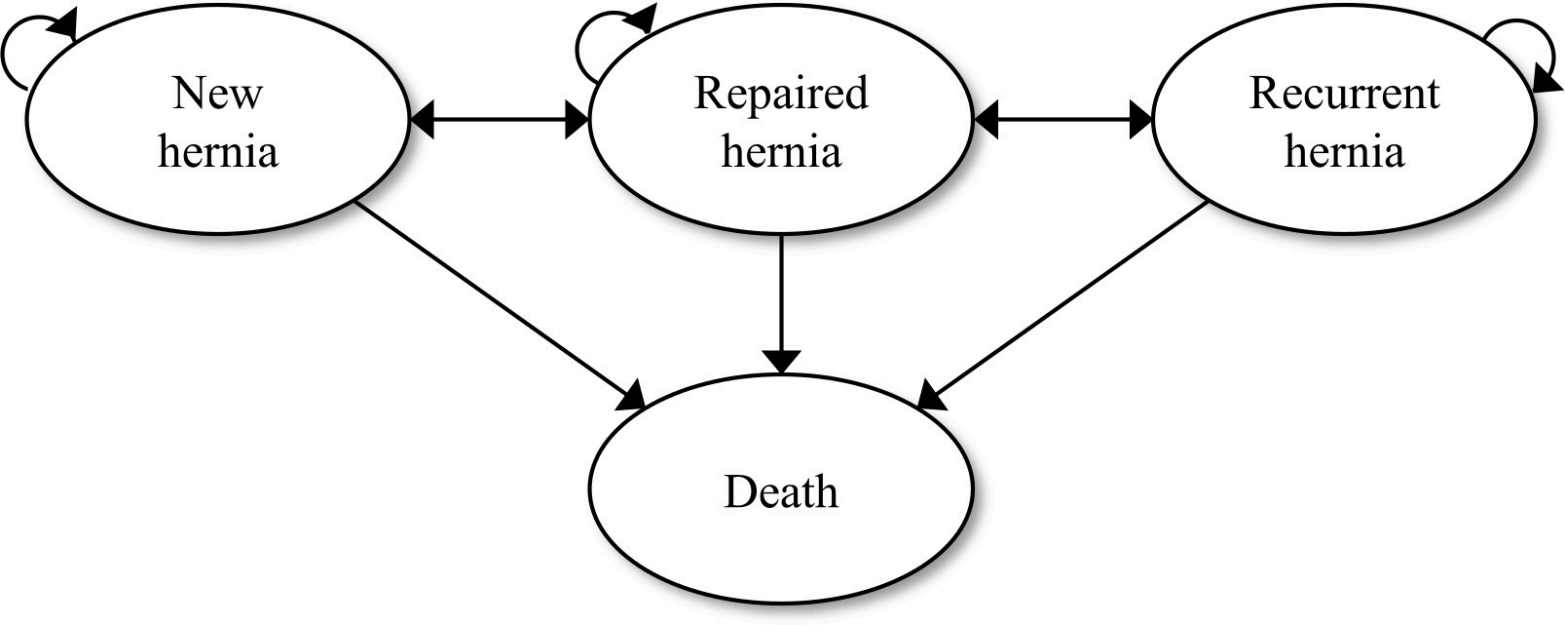
Markov model. The model includes four exclusive health states related to inguinal hernia: new hernia (primary hernia that has not previously been treated), recurrent hernia (hernia that reappears after initial surgical repair), repaired hernia (hernia that has been surgically treated and is in a post-treatment state), and death. If they receive treatment, they transition to the ’repaired’ state. From there, they may either remain in that state or move to the ’recurrent’ state or back to the ’new hernia’ state in subsequent cycles, based on the risk of recurrent and contralateral hernias, as indicated by arrows in the diagram. All health states can ultimately lead to the absorbing state of death, influenced by the risks of WHO’s age-specific mortality and premature death without surgery.

A Markov cohort model was developed in Microsoft Excel, capturing the clinical care trajectory of male adults with symptomatic inguinal hernia seeking care at district-level hospitals. Simulated transitions were depicted between four health states representing patients with new or recurrent hernia, their postoperative status, and death (Fig 1). The cycle length was one year, and a time horizon of 10 years was considered adequate for assessing the long-term effects of the condition. This duration aligns with those employed for evaluating the clinical risks of contralateral [15] and recurrent hernias [16]. A discount rate of 3% and half-cycle correction were applied. The study followed the ISPOR guideline Consolidated Health Economic Evaluation Reporting Standards (CHEERS) checklist (S1 Table).

### Health outcomes

Disability-adjusted life years (DALYs), a common health outcome measure in disease burden studies [2], were used in the current study. The equations for DALYs are provided below:

DALY = years lived with disability (YLD) + years of life lost (YLL)

YLD = disability weight (DW) × remaining life expectancy at time of surgery

YLL = risk of early death without surgery × remaining life expectancy at time of surgery

Disability weights, ranging from 0 (no disability) to 1 (worst possible disability), measure the severity of the condition. Symptomatic new or recurrent hernia patients may experience mild, moderate, or severe pain. After surgery, patients may experience relief or continue to endure chronic mild pain. Pain levels, assessed using the validated Inguinal Pain Questionnaire (IPQ) [17], were collected before and one year after surgery in a prior clinical trial [12]. These IPQ scores were converted into DWs according to the Global Burden of Disease Study 2017 [18] (S2 Table). The use of pain levels aligns with the GBD methodology, which considers pain a crucial factor in estimating disability and quality of life impacts [18]. Remaining life expectancy at the time of surgery was estimated using World Health Organization (WHO) country-specific life tables [19].

The risk of early death without surgery was estimated based on the risk of complications without surgery, such as incarceration or strangulation, and the associated mortality rate, as shown in the following equation:

Risk of early death without surgery = risk of complications without surgery × risk of mortality from complications

Previous studies estimated the risk of complications for males under 65 years of age to be between 0.0018 and 0.0056 [20, 21]. The mortality rate of untreated inguinal hernia was estimated to be between 0.06 to 0.22 [22, 23], depending on the delay in the required medical intervention, and was assumed to be 20 per cent in the current study.

### Costs

The cost of a standard operation conducted by each type of surgical provider was assessed from a healthcare system perspective. This analysis considered direct costs encompassing materials, medicines, human resources, capital expenditures, and overhead expenses. It also accounted for additional treatment costs related to postoperative complications, including excessive pain (5.22% in the AC group vs. 4.39% in the MD group), impaired wound healing (6.96% vs. 7.89%), wound infection (5.22% vs. 3.51%), and hematoma or other conditions requiring reoperations (1.74% vs. 0.88%) [12]. The resources and associated cost calculations were elaborated upon in S2 Table. All costs were converted to USD using an exchange rate of 1 USD = 7.38 Sierra Leonean Leone (SLL) [24] and adjusted to 2023 values using health-related consumer price indexes [25] to account for inflation. A detailed breakdown of cost inputs to the model is presented in S4 Table.

### Cost-effectiveness analysis

We assessed the costs and health outcomes of hernia repair performed by ACs and MDs over a 10-year period. Comparisons were made with both no treatment and between the two provider types. This approach was driven by a scarcity of specialist general surgeons, coupled with the widespread prevalence of untreated inguinal hernia patients in the country [4]. The incremental cost-effectiveness ratio (ICER) was calculated as the cost per DALY averted. Following WHO guidelines, the cost-effectiveness was determined using a willingness-to-pay (WTP) threshold set at three times the gross domestic product (GDP) per capita of Sierra Leone [26], which amounted to 3 x USD 476 in 2022 [27]. The intervention is considered cost-effective if the ICER is below this threshold and very cost-effective if the ICER is below the GDP per capita. Model inputs for this analysis are detailed in Table 1. Baseline characteristics, such as starting age, sex, and pre-operative pain levels, are defined as in the previous clinical trial [12]. We also assessed the cost-effectiveness of the surgical procedure in subgroups stratified by pre-operative pain levels, specifically moderate (IPQ 4–5) and severe (IPQ 6–7), to explore the impact of patient heterogeneity.

**Table 1:** Inputs for cost-effectiveness analysis.

Parameters		Values	Sources
Starting age (years)		40	
Time horizon (years)		10	
Cost per procedure (2023 values in USD)			
	ACs	149	
	MDs	163	
Risk of recurrent hernia (%)			
	ACs	0.92	[12]
	MDs	6.86	[12]
Risk of contralateral hernia (%)		0.38	[15]
Risk of premature death without surgery (%)		0.11	[22, 23]
Discount rate (%)		3	
Half-cycle correction		Yes	

*AC* Associate Clinician; *MD* Medical Doctor.

### Sensitivity analysis

Both deterministic and probabilistic sensitivity analyses were performed to assess the impact of uncertainty in model input values on the ICER. One-way deterministic sensitivity analysis (DSA) was employed, involving the systematic variation of one input parameter at a time within plausible limits (± 20% in the current study) to comprehend isolated effects. Discount rates of 0% and 3.5%, as well as the model with and without half-cycle correction, were also tested. Additionally, a probabilistic sensitivity analysis (PSA) was conducted by simultaneously varying all input values using a distribution function to capture the collective effects. The analysis was repeated for 1,000 simulations. Stochastic values obtained were used to construct the cost-effectiveness plane and cost-effectiveness acceptability curve. Detailed input values for the analyses are presented in Table 2.

**Table 2:** Inputs for sensitivity analysis.

Parameters		Values		
Deterministic sensitivity analysis		Base-case	Low	High
	Discount rate (%)	3	0	3.5
	Starting age (years)	40	30	50
	Cost per procedure			
	ACs	149	120	179
	MDs	163	130	195
	DWs			
	Mild pain (IPQ 2–3)	0.0110	0.0088	0.0132
	Moderate pain (IPQ 4–5)	0.1140	0.0912	0.1368
	Severe pain (IPQ 6–7)	0.3240	0.2592	0.3888
	Risk of recurrent hernia (%)			
	ACs	0.92	0.73	1.10
	MDs	6.86	5.49	8.2
	Risk of contralateral hernia (%)	0.38	0.30	0.46
	Risk of premature death without surgery (%)	0.11	0.09	0.13
Probabilistic sensitivity analysis		Base-case	PSA	Mean (SD)
	DWs			
	Mild pain (IPQ 2–3)	0.0110	0.0098	0.0114 (0.0040)
	Moderate pain (IPQ 4–5)	0.1140	0.1001	0.1140 (0.0200)
	Severe pain (IPQ 6–7)	0.3240	0.2796	0.3240 (0.0550)
	Risk of recurrent hernia			
	ACs	0.92	0.91	0.92 (0.0001)
	MDs	6.86	6.88	6.86 (0.0001)
	Risk of contralateral hernia	0.38	0.43	0.38 (0.0007)

*AC* Associate Clinician; *DW* Disability Weight; *MD* Medical Doctor.

### Budget impact analysis

A budget impact analysis was conducted to project the total cost of addressing the backlog of inguinal hernias among adult males in Sierra Leone. Two scenarios were explored, both involving an increase in surgical care through task sharing between ACs and MDs:

1. Expansion of the current repair rate with cumulative budget ceilings over the next 10 years (USD 10 million, USD 50 million, and USD 100 million).
2. Elimination of the backlog of inguinal hernias by 2033 with an open budget.

The prevalence of inguinal hernia in adult males in the country was estimated at 8.6% [4], equating to approximately 220,748 patients in 2023. The annual incidence rate of 1,250 per 100,000 [4] was projected for subsequent years, and a contemporary repair rate of 470 per 100,000 population [4] was utilized in the analysis.

A previous clinical trial demonstrated that task-sharing between MDs and ACs for hernia repair is both safe and effective [12]. This approach helps address the surgical workforce shortage in the country. For our analysis, we used the current task-sharing proportions of 68% for MDs and 32% for ACs [7].

In addition to the costs associated with surgical operations, the analysis incorporated expenses for training and facility renovation, following consultation with local clinical experts. No discounting was applied in the analysis. A one-week course of surgical training on open mesh repair, as detailed in the previous study [12], was considered, with an estimated cost of around USD 2,000 per trainee, covering travel, accommodation, equipment, and supplies. The renovation cost per facility was estimated at USD 12,000 with three surgical providers per facility [7].

We also performed a budget impact sensitivity analysis, systematically adjusting individual cost inputs by ±20%, excluding postoperative complication-related treatment costs, varying the surgical workforce per facility between 2 and 4, and enhancing provider productivity to 5 hernia repairs per day. The impact on the cumulative 10-year budget was then calculated.

### Model validation

Face validity was ensured through consultations with clinical experts throughout the study period. Internal validity, including equation reviews and systematic code walk-throughs, was conducted. Additionally, the model was validated against a comparable study conducted in Ghana (27), which involved a similar population and used analogous model structures.

### Ethics statement

The protocol for the clinical trial was approved by the Sierra Leone Ethics and Scientific Review Committee (SLESRC-ERC: 22/05/17). Written or thumb-printed informed consent was obtained from all study participants prior to enrolment (23/10/17 to 02/02/18). The Swedish Ethical Review Authority has approved data management and analysis in Sweden (2024-02758-01). This study utilized cohort-level estimates from pseudonymized data from the clinical trial [12].

## Results

### Cost-effectiveness analysis

An adult male with a primary reducible inguinal hernia was estimated to have 0.75 DALY without surgery over the next 10 years. Open mesh repair, either performed by ACs or MDs, could reduce the risk to 0.12 and 0.09, respectively, with corresponding estimated surgical care costs of USD 165 and USD 255 (Table 3). The cost per DALY averted for the operation was USD 250 (AC) and USD 411 (MD) compared to no treatment. Both ICER values were below the country’s GDP per capita of USD 476, indicating high cost-effectiveness in both provider groups. Between the two provider groups, the operation appeared to be more cost-effective in the AC group, with a lower cost of USD 90 for a health gain of 0.04 DALYs averted. The ICER values were lower for more severe patient groups. For patients with moderate pre-operative pain (IPQ 4–5), the ICER was USD 178 for ACs and USD 293 for MDs. For those with severe pre-operative pain (IPQ 6–7), the ICER was USD 64 for ACs and USD 110 for MDs.

**Table 3:** Cost-effectiveness of mesh repair by different providers in Sierra Leone.

Interventions	No hernia repair	Hernia repair by MDs (vs. no repair)	Hernia repair by ACs	
			(vs. no repair)	(vs. MD)
Base-case				
Total cost ($)	0	255	165	
Total DALYs	0.75	0.12	0.09	
Incremental cost ($)	-	255	165	-90
Incremental DALYs averted	-	0.62	0.66	0.04
ICER ($/DALY averted)	-	411	250	Dominates
Sub-group with moderate pre-operative pain (IPQ 4–5)				
Total cost ($)	0	255	165	
Total DALYs	1.05	0.18	0.13	
Incremental cost ($)	-	255	165	-90
Incremental DALYs averted	-	0.87	0.92	0.05
ICER ($/DALY averted)	-	293	178	Dominates
Sub-group with severe pre-operative pain (IPQ 6–7)				
Total cost ($)	0	255	165	
Total DALYs	2.9	0.59	0.35	
Incremental cost ($)	-	255	165	-90
Incremental DALYs averted	-	2.32	2.56	0.24
ICER ($/DALY averted)	-	110	64	Dominates

*AC* Associate Clinician; *DALY* Disability-adjusted Life Year; *ICER* Incremental Cost-effectiveness Ratio; *IPQ* Inguinal Pain Questionnaire; MD Non-specialist Medical Doctor.

### Sensitivity analysis

**Fig 2:**
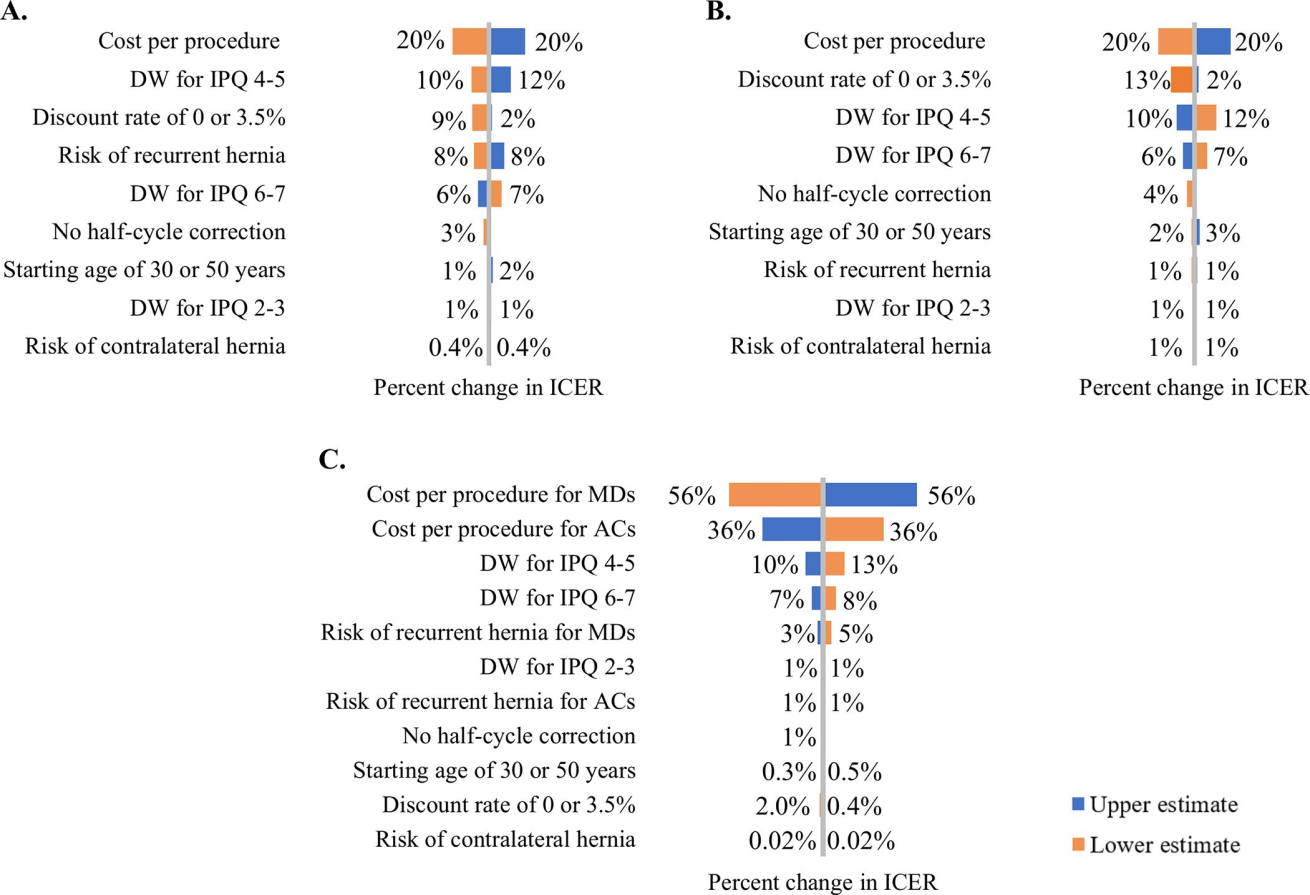
Deterministic sensitivity analysis of Incremental Cost-effectiveness Ratios (ICERs). *AC* Associate Clinician; *DW* Disability Weight; *IPQ* Inguinal Pain Questionnaire; *MD* Medical Doctor. The tornado diagram displays the percent decrease or increase in ICER values for hernia repair against various comparator groups, resulting from variations in each model input. The upper and lower estimates represent the range of variability in the data, indicating the highest and lowest values for each parameter analysed. (A) hernia repair by MDs vs. no repair, (B) hernia repair by ACs vs. no repair, (C) hernia repair by ACs vs. hernia repair by MDs.

Deterministic sensitivity analysis in Fig 2 shows that the base-case ICER value was most sensitive to the cost per procedure across all three comparisons. This was followed by disability weights (DWs) for moderate pain (IPQ 4–5), severe pain (IPQ 6–7), the risk of recurrent hernia in the MD group, and the application of no discounting. Despite these variations, the ICER remained below the threshold value.

**Fig 3:**
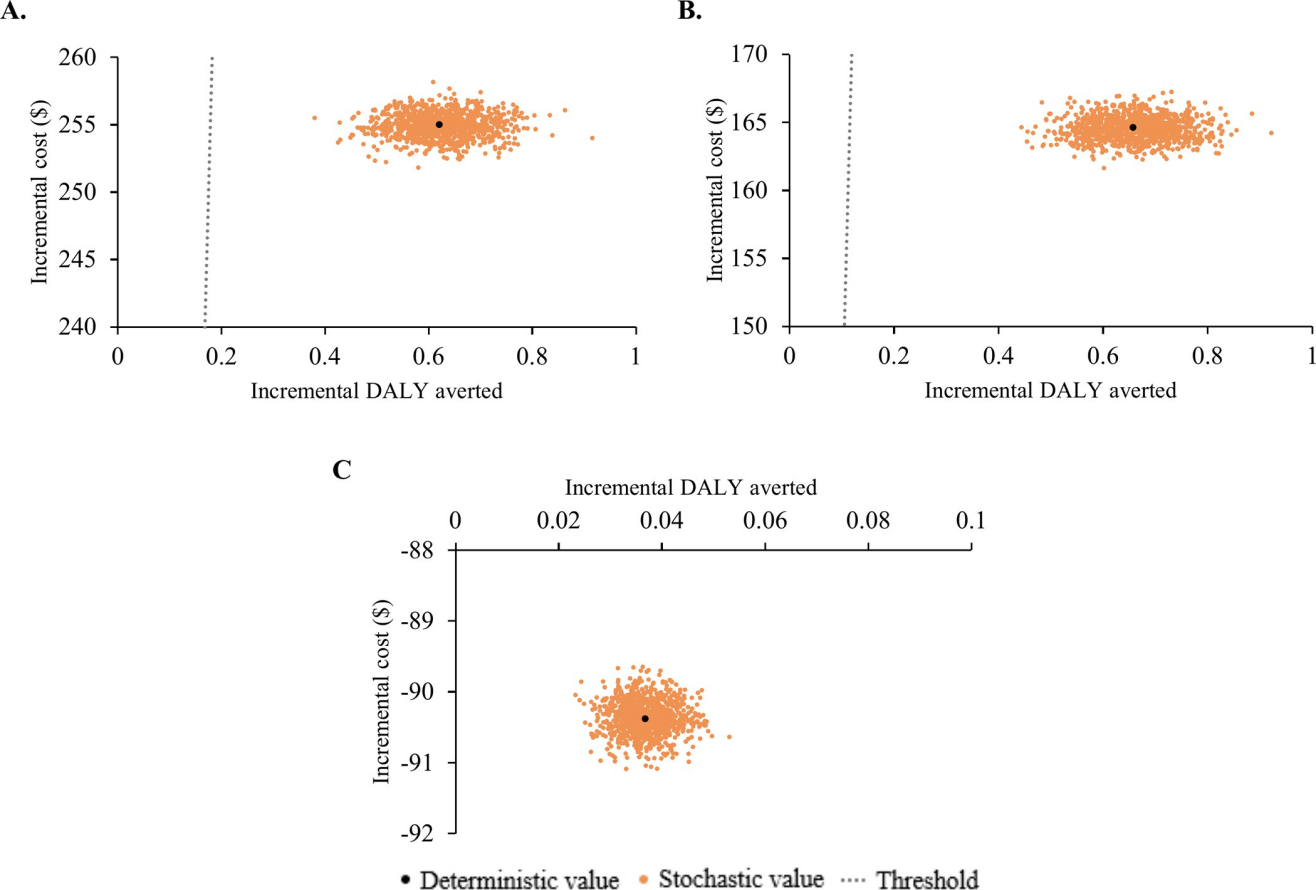
Stochastic sensitivity analysis of Incremental Cost-effectiveness Ratios (ICERs). *AC* Associate Clinician; *DALY* Disability-adjusted Life Year; *MD* Medical Doctor. The scatter plot illustrates deterministic and stochastic values of incremental cost (in USD millions) and incremental DALYs averted (in thousands) from hernia repair against various comparator groups within a cost-effectiveness plan, accounting for variations in model inputs. (A) hernia repair by MDs vs. no repair, (B) hernia repair by ACs vs. no repair, (C) hernia repair by ACs vs. hernia repair by MDs.

Probabilistic sensitivity analysis in Fig 3 demonstrates the model’s robustness to parameter uncertainty, with values clustering around the base-case ICER. For ACs or MDs vs. no hernia, the values stay below the threshold line in the northeast quadrant (indicating higher costs with more DALYs averted) of the cost-effectiveness plane. For ACs vs. MDs, they fall within the southeast quadrant (dominant, indicating lower costs with more DALYs averted), supporting our findings.

**Fig 4:**
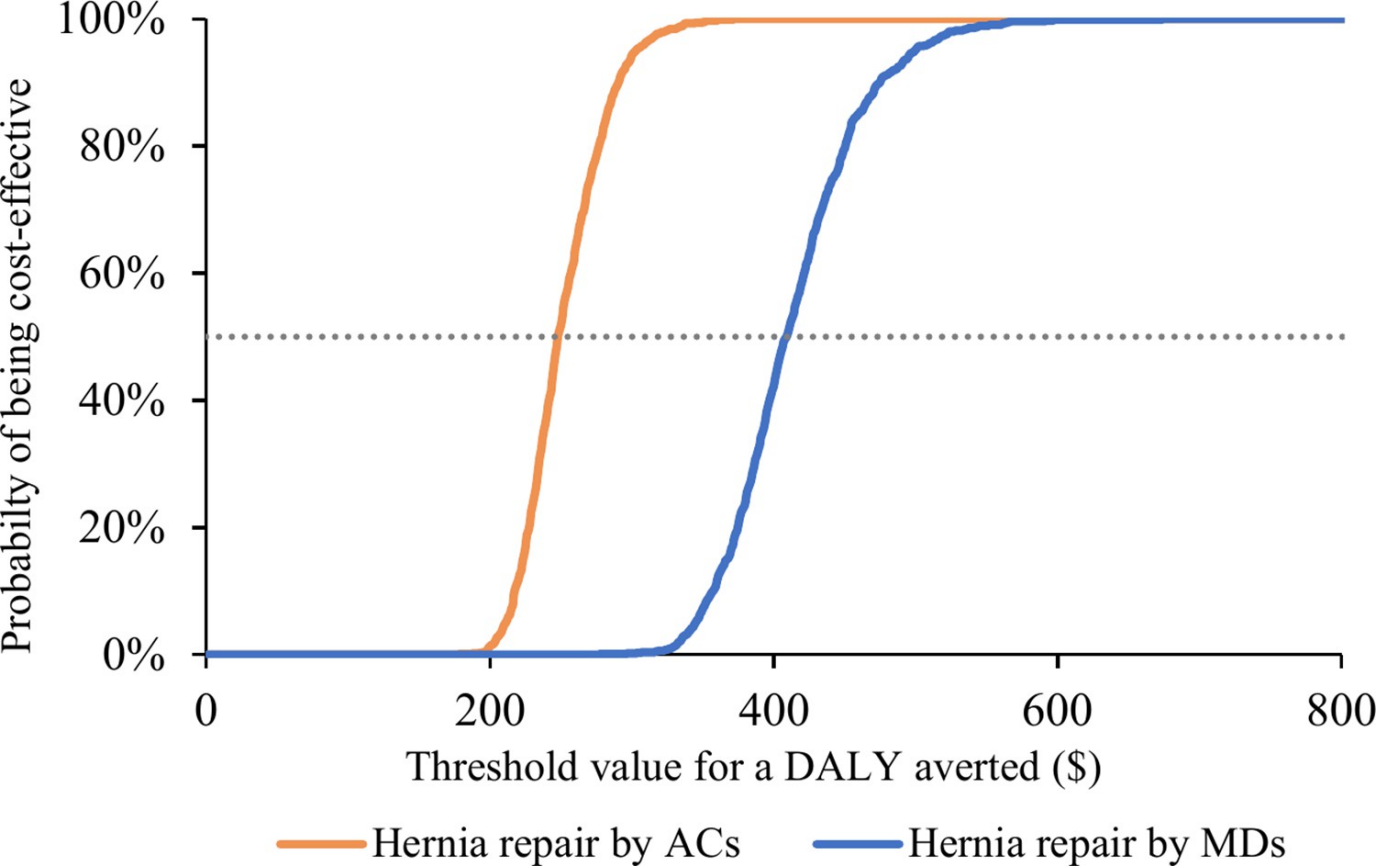
Cost-effectiveness acceptability curves. *AC* Associate Clinician; *DALY* Disability-adjusted Life Year; *MD* Medical Doctor. The line graph depicts the probability of cost-effectiveness for inguinal hernia repair by ACs and MDs vs. no repair at various threshold values for a DALY averted ($).

The cost-effectiveness acceptability curve in Fig 4 shows that the operation becomes cost-effective at approximately USD 248 per DALY averted for ACs and USD 409 for MDs. The probability reaches 100% at USD 372 for ACs and USD 674 for MDs, both below our threshold value.

### Budget impact analysis

**Fig 5:**
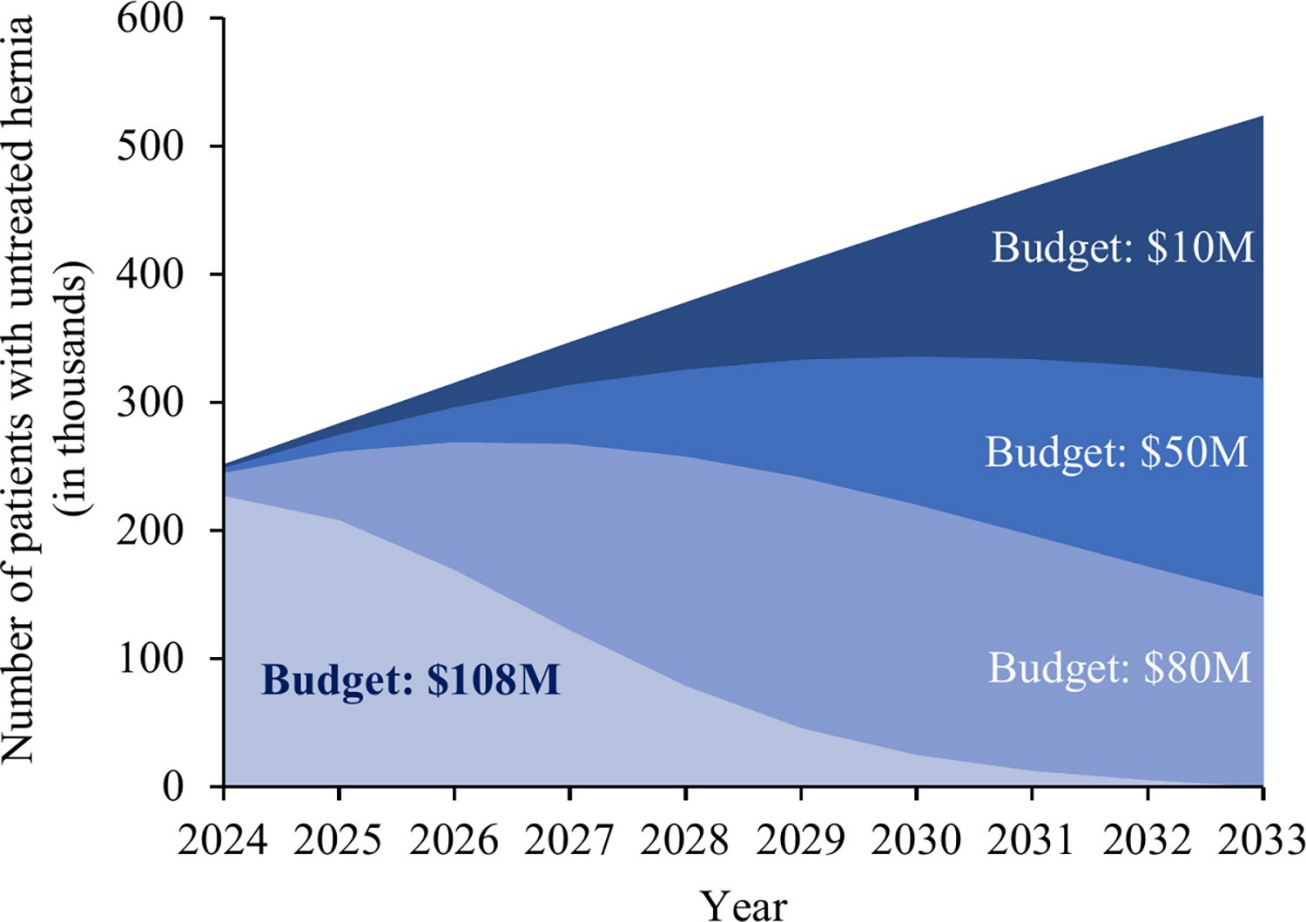
Inguinal hernia repair expansion for adult males in Sierra Leone (2024–2033). The graph illustrates the budget impact on untreated cases (in thousands) over the next decade.

Expanding inguinal hernia repairs with mesh for adult males in Sierra Leone through task-sharing between ACs and MDs was analyzed over a 10-year period under different budget scenarios. The cumulative costs were estimated by increasing the current repair rate through task-sharing within annual budget limits, including expenses for training additional surgical providers, renovating facilities, and conducting surgical operations. The total estimated costs were USD 10 million to address 9% of the backlog, USD 50 million to address 45%, and USD 80 million to address 74%. A budget of USD 50 million would minimize backlog accumulation (Fig 5). Clearing the entire backlog of approximately 578,000 patients would cost around USD 108 million. This would necessitate the training of 2,100 additional surgical providers. The estimated budget could be reduced by increasing the productivity of surgical providers, with additional savings from workforce distribution, facility renovation, and training costs (S1 Fig). Reducing post-operative complication risks could further lower the total budget by about 2%.

## Discussion

This study assessed the cost-effectiveness of inguinal hernia repair in adult males in Sierra Leone. Using the WHO-recommended threshold of GDP per capita (USD 476) [26], the operation performed by either ACs or MDs was deemed very cost-effective compared to no surgical repair, consistent with earlier studies in Ghana [28, 29]. Alternatively, considering opportunity cost as a threshold, our ICER values (USD 250 for ACs and USD 411 for MDs) fall within the estimated range for Sierra Leone, USD 98 to 1,845 (adjusted to 2023 value) [30]. Threshold selection requires context-specific and nuanced discussions, particularly when evaluating healthcare interventions in resource-limited settings.

Within subgroups of patients experiencing moderate pre-operative pain, hernia repair demonstrated enhanced health gains for the same operational costs in each provider group. This effect was further amplified for patients with severe pre-operative pain, indicating a higher degree of cost-effectiveness. This finding suggests a potential translation into policy, advocating for the prioritization of hernia patients based on the severity of their condition, especially when faced with budget constraints.

Between the two types of providers, choosing ACs for the operation appears to yield specific cost savings (USD 90) with similar health gains, attributed to their lower recurrence rates and lower salaries compared to MDs. This conclusion may be exaggerated, as the recurrence rate in the MD group was unusually high, while the AC group’s rate was comparable to findings in similar country contexts [12]. One possible explanation is that MDs receive basic surgical training during their internship, whereas ACs undergo a more structured postgraduate surgical training program. Implementing a systematic surgical training program for MDs may be necessary to enhance their surgical skills.

Exploring the potential expansion of task-sharing with ACs and MDs as a strategy to address unmet surgical needs, this study projected a 10-year budget of USD 104 million (equivalent to 14.5% of Sierra Leone’s 2022 health budget [31]) to clear the entire backlog of approximately 578,000 patients. This estimate includes training an additional 2,100 providers, assumed to have completed their general training programs lasting 3 to 6 years for ACs and MDs, along with facility renovation. Including both ACs and MDs in the budget estimates reflects the need to enhance the overall quality of surgical care and to address the varying levels of surgical training among providers. Scaling up to this extent within a 10-year timeframe would be challenging in Sierra Leone, further compounded by the earmarked nature of the country’s healthcare funds for specific disease areas, relying on external donors [31].

Despite the challenges posed by limited health funds, expanding task-sharing can be approached strategically. Key opportunities include realizing economies of scale by sharing resources with other surgical services, such as medical equipment and facilities, while leveraging the enhanced surgical skills of ACs and MDs. Enhancing collaboration with existing healthcare programs and exploring external funding options can also help address budget constraints. Increasing the productivity and density of surgical providers at each facility, as suggested by our budget impact sensitivity analysis, can further improve efficiency. Additionally, this initiative could generate significant societal benefits by reducing productivity losses at both the household and national levels.

In addition to efforts to expand the surgical workforce, strategies should be developed to address the prevalent maldistribution of healthcare personnel between urban and rural areas, estimated at 8 to 1 [7, 14]. District-level hospitals, which play a crucial role in providing care to rural communities, are key to improving access to surgical services in these areas. Both ACs and MDs show a more balanced distribution ratio than specialist surgeons [14], with ACs being more likely to be retained in rural posts. Previous studies also observed that operations conducted by ACs were comparable to those by MDs in terms of safety and effectiveness [6, 12]. Therefore, engaging ACs and MDs in hernia repair particularly at district hospitals, supported by policies incentivizing service in rural communities, could be a potential investment with favourable returns for rural access and service quality.

Moreover, financial accessibility is of equal significance to healthcare access [32]. Prior research has highlighted that a major barrier to seeking medical assistance for hernias is the inability to afford medical bills [4]. This study, adopting a healthcare system perspective, did not delve into out-of-pocket payments or the broader societal costs associated with productivity loss due to this medical condition. Incorporating these considerations in future research would enhance the discourse on the diverse challenges associated with hernia treatment and, in a broader aspect, contribute to the establishment of a sustainable healthcare financing system for the country.

### Strengths and limitations

This study has several strengths. Firstly, it utilizes data from a rigorously controlled randomized clinical trial, which enhances the reliability of the data. Secondly, the model’s flexibility and robustness allowed for the analysis of various scenarios involving different mixes of associate clinicians and medical doctors, providing valuable insights into the cost-effectiveness of task-sharing interventions. Lastly, the study accounted for critical clinical events, such as recurrence risks and contralateral hernias, which significantly influence the long-term course of the disease.

However, the study also has limitations. The use of a single randomized trial in a district hospital setting may affect the generalizability of the findings while it provides a specific context for the analysis. Additionally, although the healthcare system perspective was adopted to focus on direct costs, this approach may overlook broader economic impacts, such as societal productivity losses. Including these indirect costs could potentially make the intervention even more cost-effective, as it would highlight additional economic benefits beyond the healthcare setting. Finally, the calculation of DALYs was based solely on patients’ reported pain levels, which may not comprehensively reflect the multidimensional aspects of health.

Further research on local insights regarding supportive quality control measures for effective task sharing implementation would be beneficial to strengthen the policy.

## Conclusions

This study shows the cost-effectiveness of open mesh inguinal hernia repair for adult males, whether performed by ACs or MDs in Sierra Leone. Notably, in certain African countries like Ghana, ACs are not currently recognized as a cadre of healthcare workforce for surgical task sharing. However, this study reveals that ACs are more cost-effective than MDs, provided that there is assurance of quality training and the presence of supportive policies and supervision. This suggests that considering task-sharing with ACs should be contemplated even in contexts where their utilization is not presently permitted. Moreover, conducting systematic surgical training for MDs could further enhance their surgical skills, allowing the country to optimize all available human resources. Task-sharing with ACs could be a potential solution to address the ongoing shortage of surgical professionals and the significant backlog of inguinal hernias, especially in rural areas. This underscores the importance of supporting and implementing task-sharing initiatives as a key policy approach to enhance accessibility and efficiency in surgical care, especially in resource-constrained settings. Future research could delve deeper into the potential scalability and long-term sustainability of such strategies. This study adds to the growing body of evidence advocating for innovative approaches to bridge healthcare gaps, and its implications extend beyond inguinal hernia repair to broader considerations of healthcare delivery in low-resource settings.

## Supporting information

S1 Checklist Inclusivity in global research. (DOCX)

S1 Fig Deterministic sensitivity analysis of the estimated budget of addressing the backlog of inguinal hernia among adult men in Sierra Leone by 2033. The tornado chart shows percentage changes in the total cost based on input variations in the budget impact analysis.
(TIFF)

S1 Table Consolidated health economic evaluation reporting standards (CHEERS) 2022 checklist. (DOCX)

S2 Table Mapping inguinal pain questionnaire (IPQ) scores to disability weights (DWs) in alignment with the global burden of disease study 2017. (DOCX)

S3 Table Measurement of resources and costs. *AC* Associate Clinician; *MD* Medical Doctor; *OR* Operating Room. *Corresponds to a 5-day working week for elective surgical procedures, 52 weeks/year. †Estimated maximal capacity for 3 operating tables each with 5 operations/day. ‡The surgical care (operating theatre and wards) was estimated to use 50% of water and electricity expenses, 100% of the fuel and 20% of the administrative resources. ⸸Estimated maximal capacity for appointments with 15 minutes per patient leading to maximal capacity of 32 patients per day per room at the outpatient ward with 4 examination rooms.
(DOCX)

S4 Table Cost breakdown by resource items and surgical provider types. *AC* Associate Clinician; *MD* Medical Doctor.
(DOCX)
