# Supplementary figures and images for: Economic evaluation of expanding inguinal hernia repair among adult males in Sierra Leone
Source: PLOS Glob Public Health. 2024 Dec 12;4(12):e0003861. doi: 10.1371/journal.pgph.0003861 (PMC11637271; doi:10.1371/journal.pgph.0003861)

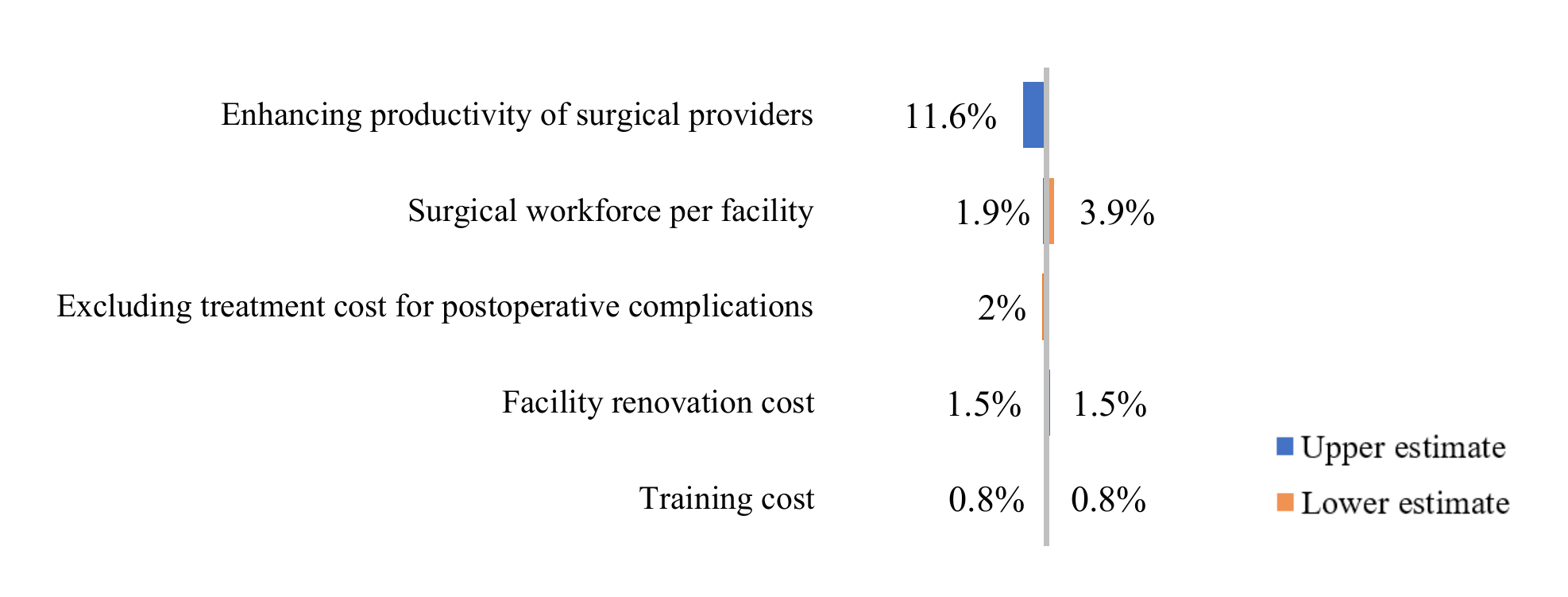

Supplement: S1 Fig — The tornado chart shows percentage changes in the total cost based on input variations in the budget impact analysis. (TIFF) [file pgph.0003861.s002.tiff]
