# Supplementary material for: Economic evaluation of expanding inguinal hernia repair among adult males in Sierra Leone
Source: PLOS Glob Public Health. 2024 Dec 12;4(12):e0003861. doi: 10.1371/journal.pgph.0003861 (PMC11637271; doi:10.1371/journal.pgph.0003861)
Supplement: S1 Table — (DOCX) [file pgph.0003861.s003.docx]

S1 Table. Consolidated health economic evaluation reporting standards (CHEERS) 2022 checklist

|  | **Item** | **Guidance for reporting** | **Reported in section** |
| --- | --- | --- | --- |
| **Title** | | | |
| Title | 1 | Identify the study as an economic evaluation and specify the interventions being compared. | Page 1 |
| **Abstract** | | | |
| Abstract | 2 | Provide a structured summary that highlights context, key methods, results and alternative analyses. | Page 2 |
| **Introduction** | | | |
| Background and objectives | 3 | Give the context for the study, the study question and its practical relevance for decision making in policy or practice. | Page 3-4 |
| **Methods** | | | |
| Health economic analysis plan | 4 | Indicate whether a health economic analysis plan was developed and where available. | NA |
| Study population | 5 | Describe characteristics of the study population (such as age range, demographics, socioeconomic, or clinical characteristics). | Page 4 |
| Setting and location | 6 | Provide relevant contextual information that may influence findings. | Page 4 |
| Comparators | 7 | Describe the interventions or strategies being compared and why chosen. | Page 4 |
| Perspective | 8 | State the perspective(s) adopted by the study and why chosen. | Page 6 |
| Time horizon | 9 | State the time horizon for the study and why appropriate. | Page 5 |
| Discount rate | 10 | Report the discount rate(s) and reason chosen. | Page 5 |
| Selection of outcomes | 11 | Describe what outcomes were used as the measure(s) of benefit(s) and harm(s). | Page 6 |
| Measurement of outcomes | 12 | Describe how outcomes used to capture benefit(s) and harm(s) were measured. | Page 6 |
| Valuation of outcomes | 13 | Describe the population and methods used to measure and value outcomes. | Page 6 |
| Measurement and valuation of resources and costs | 14 | Describe how costs were valued. | Page 6-7 (S3 and S4 Table) |
| Currency, price date, and conversion | 15 | Report the dates of the estimated resource quantities and unit costs, plus the currency and year of conversion. | Page 6-7 |
| Rationale and description of model | 16 | If modelling is used, describe in detail and why used. Report if the model is publicly available and where it can be accessed. | Page 5 |
| Analytics and assumptions | 17 | Describe any methods for analysing or statistically transforming data, any extrapolation methods, and approaches for validating any model used. | Page 6 |
| Characterizing heterogeneity | 18 | Describe any methods used for estimating how the results of the study vary for sub-groups. | Page 7 |
| Characterizing distributional effects | 19 | Describe how impacts are distributed across different individuals or adjustments made to reflect priority populations. | Page 7 |
| Characterizing uncertainty | 20 | Describe methods to characterize any sources of uncertainty in the analysis. | Page 8 |
| Approach to engagement with patients and others affected by the study | 21 | Describe any approaches to engage patients or service recipients, the general public, communities, or stakeholders (e.g., clinicians or payers) in the design of the study. | NA |
| **Results** | | | |
| Study parameters | 22 | Report all analytic inputs (e.g., values, ranges, references) including uncertainty or distributional assumptions. | Page 7-9 (Table 1 and 2) |
| Summary of main results | 23 | Report the mean values for the main categories of costs and outcomes of interest and summarise them in the most appropriate overall measure. | Page 12-13 (Table 3) |
| Effect of uncertainty | 24 | Describe how uncertainty about analytic judgments, inputs, or projections affect findings. Report the effect of choice of discount rate and time horizon, if applicable. | Page 13-15 (Fig 2, 3 and 4) |
| Effect of engagement with patients and others affected by the study | 25 | Report on any difference patient/service recipient, general public, community, or stakeholder involvement made to the approach or findings of the study | NA |
| **Discussion** | | |  |
| Study findings, limitations, generalizability, and current knowledge | 26 | Report key findings, limitations, ethical or equity considerations not captured, and how these could impact patients, policy, or practice. | Page 16-18 |
| **Other relevant information** | | | |
| Source of funding | 27 | Describe how the study was funded and any role of the funder in the identification, design, conduct, and reporting of the analysis | Reported to the journal |
| Conflicts of interest | 28 | Report authors conflicts of interest according to journal or International Committee of Medical Journal Editors requirements. | Reported to the journal |
