# Supplementary material for: Economic evaluation of expanding inguinal hernia repair among adult males in Sierra Leone
Source: PLOS Glob Public Health. 2024 Dec 12;4(12):e0003861. doi: 10.1371/journal.pgph.0003861 (PMC11637271; doi:10.1371/journal.pgph.0003861)
Supplement: S2 Table — (DOCX) [file pgph.0003861.s004.docx]

S2 Table. Mapping inguinal pain questionnaire (IPQ) scores to disability weights (DWs) in alignment with the global burden of disease study 2017.

| **Description of pain** | | **IPQ score** | **DW** |
| --- | --- | --- | --- |
| No pain | | 1 | 0 |
| Mild pain | | | |
|  | Pain present, but can easily be ignored | 2 | 0.011 |
|  | Pain present, cannot be ignored but does not interfere with daily activities | 3 |  |
| Moderate pain | | | |
|  | Pain present, cannot be ignored but interferes with a concentration on chores and daily activities/ | 4 | 0.114 |
|  | Pain present, cannot be ignored but interferes with most activities | 5 |  |
| Severe pain | | | |
|  | Pain present, cannot be ignored, necessitates bed rest | 6 | 0.324 |
|  | Pain present, cannot be ignored, prompt medical advice sought | 7 |  |
