# Supplementary material for: Economic evaluation of expanding inguinal hernia repair among adult males in Sierra Leone
Source: PLOS Glob Public Health. 2024 Dec 12;4(12):e0003861. doi: 10.1371/journal.pgph.0003861 (PMC11637271; doi:10.1371/journal.pgph.0003861)
Supplement: S3 Table — AC Associate Clinician; MD Medical Doctor; OR Operating Room. *Corresponds to a 5-day working week for elective surgical procedures, 52 weeks/year. †Estimated maximal capacity for 3 operating tables each with 5 operations/day. ‡The surgical care (operating theatre and wards) was estimated to use 50% of water and electricity expenses, 100% of the fuel and 20% of the administrative resources. ⸸Estimated maximal capacity for appointments with 15 minutes per patient leading to maximal capacity of 32 patients per day per room at the outpatient ward with 4 examination rooms. (DOCX) [file pgph.0003861.s005.docx]

S3 Table. Measurement of resources and costs.

| **Resource items** | | **Equations for cost estimation** | **Sources** |
| --- | --- | --- | --- |
| Medicines and materials | | | |
| Medicines | Pre-, inter and postoperative medication | No. of items per standard operation x price per item | Participating hospital staff, IDA April 2017 |
| Materials | Materials for preoperative, inter-operative, and postoperative examination | No. of items per standard operation x price per item | Purchases made for the study, IDA April 2017 |
| Staff time | | | |
| Operations | Costs for one MD respective one AC | Annual salaries for MDs respective ACs / 52 x 5* / 5 operations per day per table | Salaries from Ministry of Health and Sanitation of Sierra Leone |
|  | Costs for two scrub nurses and one nurse anaesthetist in the OR | Annual salary for other theatre staff / 52 x 5* / 5 operations per day per table |  |
|  | Costs for one operation technician and one cleaner | Annual salary for other theatre staff / 52 x 5* / 5 x 3† operations per day | Hospital pay roll |
| Ward care | Cost for one nurse working 12 h/d | Annual salary for a nurse / 52 x 5* / 5 x 3† operations per day | Salaries from Ministry of Health and Sanitation of Sierra Leone |
| Pre- and postoperative appointments | Cost of one MD respective on AC per 15 minutes per room | Annual salary for MDs respective ACs / 52 x 5* / 32⸸ maximum appointments per day per room | Salaries from Ministry of Health and Sanitation of Sierra Leone |
|  | Cost of one nurse per 15minutes for all 4 available rooms | Annual salary for nurse / 52 x 5* / 32 x 4⸸ |  |
| Overhead costs | | | |
| Hospital maintenance | Cost of maintenance and rehabilitation | Maintenance expenses / 52 x 5* / 5 x 3† operations per day / 2‡ | Hospital end-of-year report |
| Water | Cost of maintenance of the water supply | Hospital water expenses / 52 x 5* / 5 x 3† operations per day /2‡ |  |
| Electricity | Cost of maintenance of the solar panels and generators | Hospital electricity expenses / 52 x 5* / 5 x 3† operations per day / 2‡ |  |
| Security | Cost for one guard | Annual salary for a guard / 52 x 5* / 5 x 3† operations per day | Hospital payroll |
| Fuel | Cost of fuel | Daily fuel consumption / 5 x 3† operations per day | Fuel logs during the study |
| Cleaning supplies | Cost of cleaning supplies | Hospital cleaning supplies / 52 x 5* / 5 x 3† operations per day / 2‡ | Hospital end-of-year report |
| Administration | Cost for an accountant and a financial officer | Annual salaries / 52 x 5* / 5 x 3† operations per day / 5‡ | Hospital payroll |
| Equipment | Cost of equipment/operation if everything had been bought new.  Major equipment depreciated over 5, intermediate over 3 and minor over 1 year. | Depreciation calculates as cost / 52 x 5* x No. of years each item is depreciated over.  Day cost / No. of operations in which each item is used/operation day | Hospital staff, survey in theatre and online sources |
| Overhead costs for pre- and postoperative appointments⸹ | As all above excluding equipment | 10% of all hospital overhead costs used for the building with 4 examination rooms / 32 x 4⸸ | As all above excluding equipment |
| Capital costs | | | |
| Operating theatre | Potential costs if the theatre space used had been rented from the village of Kamakwie | Theatre space used in m^2^ x yearly cost per m^2^ in Kamakwie village / 52 x 5* / Total of 5 x 3† surgeries per day | Measurements of the building and tenants leasing a house in Kamakwie |
| Ward for patients to spend a day | Potential costs if the ward space used had been rented from the village of Kamakwie | Ward space used in m^2^ x yearly cost per m^2^ in Kamakwie village / 52 x 5* / Total of 5 x 3† surgeries per day |  |
| Administrative building | Potential costs if the administrative space used had been rented from the village of Kamakwie | Administrative space used in m^2^ x yearly cost per m^2^ in Kamakwie village / 52 x 5* / Total of 5 x 3† surgeries per day |  |
| Outpatient clinic for patient appointments | Potential costs if the ward space used had been rented from the village of Kamakwie | Ward space used in m^2^ x yearly cost per m^2^ in Kamakwie village / 52 x 5*/ Total of 32 x 4⸸ patients met on appointments per day |  |
| Other | | | |
| Costs for postoperative complications | Excessive pain | Pain medications x 2 weeks | Related sources from above. |
|  | Impaired wound healing | Additional post-operative appointment for wound care including dressing materials like gauze, tape, gloves, and saline. |  |
|  | Wound infection requiring antibiotics | Antibiotics x 1 week |  |
|  | Reoperation for hematoma and other complications. | Cost per procedure, excluding the mesh. |  |
| Meals | One meal/patient in the hospital | Cost per meal per patient | Study budget |
