# Supplementary material for: Economic evaluation of expanding inguinal hernia repair among adult males in Sierra Leone
Source: PLOS Glob Public Health. 2024 Dec 12;4(12):e0003861. doi: 10.1371/journal.pgph.0003861 (PMC11637271; doi:10.1371/journal.pgph.0003861)
Supplement: S4 Table — AC Associate Clinician; MD Medical Doctor. (DOCX) [file pgph.0003861.s006.docx]

S4 Table. Cost breakdown by resource items and surgical provider types

| **Resource items** | | **Description** | **Cost ($)** | |
| --- | --- | --- | --- | --- |
|  |  |  | **MD** | **AC** |
| Medicines and materials | | | | |
|  | Medicines | Standard use of antibiotic prophylaxis, local anaesthesia, intravenous fluids, and postoperative pain management | 12.1 | 12.1 |
|  | Perioperative materials | Lightweight commercial mesh | 14.4 | 14.4 |
|  |  | Other | 29.1 | 29.1 |
| Staff costs | | | | |
|  | Costs of staff at operating theatre, pre-and postoperative appointments, and postoperative wards | 1 MD or AC (operation time and pre-and postoperative appointments) | 22.3 | 7.7 |
|  |  | 1 nurse anaesthetist, 1 scrub nurse, 1 operation technician and cleaning staff | 14.2 | 14.2 |
|  |  | Ward staff, nurse for pre-and post-operative appointments | 1.5 | 1.5 |
|  | Overhead costs | Share of the operating theatre, ward, laboratory, administration, outpatient department and records office, cleaning and sanitation, hospital guard, maintenance, water supply, electricity, and fuel | 47.6 | 47.6 |
|  | Capital costs | Share of operating theatre, ward, and accessory buildings | 0.9 | 0.9 |
|  | Equipment | Minor | 1.5 | 1.5 |
|  |  | Intermediate | 0.3 | 0.3 |
|  |  | Major | 4.0 | 4.0 |
| Other | | | | |
|  | Meals | One meal | 12.1 | 12.1 |
| Cost per operation (in total) | | | 218.0 | 203.4 |
